# “Who Doesn’t Like Receiving Good News?” Perspectives of Individuals Who Received Genomic Screening Results by Mail

**DOI:** 10.3390/jpm11050322

**Published:** 2021-04-21

**Authors:** Annika T. Beck, Erica J. Sutton, Carolyn P. Y. Chow, Susan H. Curtis, Iftikhar J. Kullo, Richard R. Sharp

**Affiliations:** 1 Biomedical Ethics Research Program, Mayo Clinic, Rochester, MN 55901, USA; beck1477@umn.edu (A.T.B.); suttonericaj@gmail.com (E.J.S.); carochow@sas.upenn.edu (C.P.Y.C.); curtis.susan@mayo.edu (S.H.C.); 2 Division of Cardiovascular Diseases, Mayo Clinic, Rochester, MN 55901, USA; kullo.iftikhar@mayo.edu; 3 Department of Health Sciences Research, Mayo Clinic, Rochester, MN 55901, USA

**Keywords:** genomic screening, return of results, ethical, legal, social issues, genetic counseling, patient communication, neutral genetic results

## Abstract

As genomic sequencing expands to screen larger numbers of individuals, offering genetic counseling to everyone may not be possible. One approach to managing this limitation is for a genetic counselor to communicate clinically actionable results in person or by telephone, but report other results by mail. We employed this approach in a large genomic implementation study. In this paper, we describe participants’ experiences receiving genomic screening results by mail. We conducted 50 semi-structured telephone interviews with individuals who received neutral genomic screening results by mail. Most participants were satisfied receiving neutral results by mail. Participants generally had a good understanding of results; however, a few participants had misunderstandings about their genomic screening results, including mistaken beliefs about their disease risk and the comprehensiveness of the test. No one reported plans to alter health behaviors, defer medical evaluations, or take other actions that might be considered medically problematic. Reporting neutral results by mail is unlikely to cause recipients distress or generate misunderstandings that may result in reduced vigilance in following recommended preventive health strategies. Nonetheless, some individuals may benefit from additional genetic counseling support to help situate their results in the context of personal concerns and illness experiences.

## 1. Introduction

As the cost of genomic sequencing continues to decline and the number of multi-gene panels increases, new forms of genomic screening are emerging at an unprecedented rate [1]. Early experiences with genomic screening suggest that the vast majority of people who pursue sequencing will receive a neutral result (often referred to as negative, non-medically actionable, or non-pathogenic variant) that does not suggest a need for medical follow up. Although large numbers of individuals have participated in some form of genomic screening, few data are available on how individuals understand and interpret neutral results. Additionally, studies that have examined the psychosocial impact of receiving a neutral genetic test result have focused on the experiences of symptomatic patients who were pursuing a genetic diagnosis due to family history or clinical presentation, not patients pursuing genomic risk evaluation [2,3,4,5,6].

As genomic screening activities expand, some academic medical centers have elected to communicate neutral genomic screening results by mail or online instead of in person, including several large genomic studies supported by the National Institutes of Health (NIH) [7,8,9]. This is a noteworthy departure from early approaches to genomic testing, where all genomic results were typically reported in person by a genetic counselor or other genetic specialist. Providing neutral results by mail allows genetic counselors to focus their specialized skills in support of the individuals who are most significantly impacted by genomic screening.

It is unclear how individuals may understand neutral screening results or interpret the relevance of those results to their health, particularly when neutral results are reported in the absence of genetic counseling support. Of particular concern is the possibility that some people may misinterpret neutral screening results as a comprehensive assessment of their health; an invitation to be less vigilant in following preventive health recommendations; or a rationale for not following medical advice based on family history or other risk factors. To evaluate these concerns, we interviewed individuals who received neutral genomic screening results by mail. Our findings contribute much-needed data to ongoing discussions regarding best practices for reporting neutral genomic screening results.

## 2. Materials and Methods

**Figure 1:**
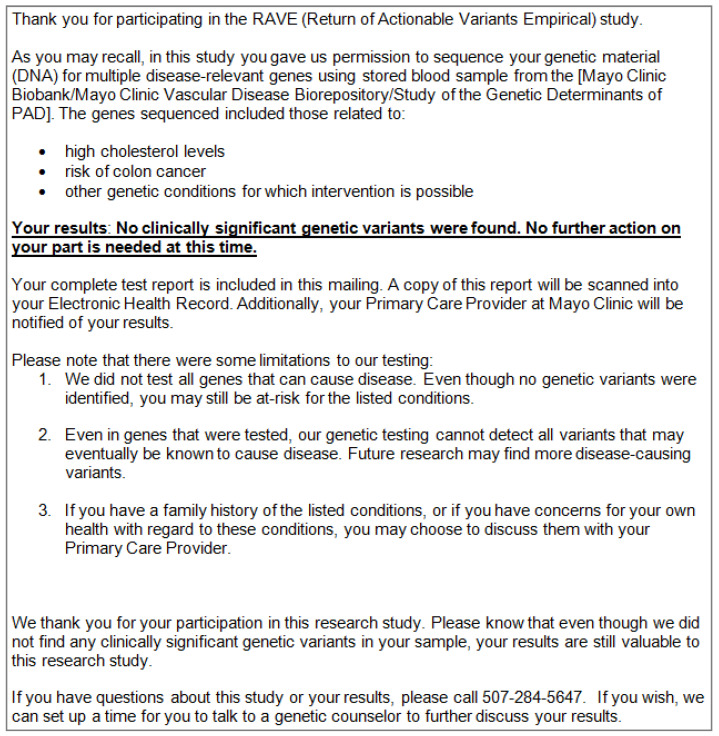
Text from the negative results letter which was distributed to participants receiving neutral results.

As part of the NIH-supported eMERGE consortium, Mayo Clinic initiated the Return of Actionable Variants Empirical (RAVE) Study, a genomic implementation study that reported pathogenic/likely pathogenic results in 68 genes and 14 actionable single-nucleotide variants to 2535 individuals [10]. Participants were not required to meet with a genetic counselor before enrolling in this study or after receiving neutral results by mail, but optional genetic counseling was available to participants at no cost [11]. Genetic counselors returned positive results (i.e., medically actionable, pathogenic or likely pathogenic variants) to participants in person or by telephone and arranged referrals to medical specialists as appropriate. Participants with neutral results (benign and likely benign variants, as well as variants of uncertain significance) received a mailed packet that included a results letter and a copy of their laboratory report (see Figure 1). The letter summarizing neutral results was developed by the research team, in consultation with genetic counselors and a Community Advisory Board supported by Mayo Clinic’s Center for Individualized Medicine [12]. Additional information about the RAVE study is available elsewhere [10,13].

Research staff invited RAVE study participants who had received a neutral results letter and completed a baseline psychosocial survey at enrollment to participate in this IRB-approved interview study [14]. Midway through recruitment, we began oversampling men, younger participants, and individuals with lower levels of genetic literacy to increase the diversity of participants.

From September to December 2017, two qualitative researchers (EJS, ATB) used a semi-structured, open-ended interview guide to conduct telephone interviews. All interviews were audio-recorded and transcribed verbatim. Interviewers wrote field notes after each interview and regularly compared field notes to adjust the interview guide to maximize the effectiveness of future interviews and aid in preliminary analysis.

All transcripts were reviewed for accuracy. Two analysts read and discussed a subset of the transcripts (ATB, EJS) and, in conjunction with field note summaries, created an initial codebook. The codebook was revised in collaboration with two additional analysts (SHC, CPYC). These four analysts then coded six transcripts independently to refine the codebook and establish reliability across coders. Thereafter, analysts coded transcripts in pairs and consensus codes were entered in QSR International’s NVivo 11 Software (2015 release) to facilitate data analysis [15]. Summary memos were prepared for primary codes and two analysts (ATB, EJS) identified and described major themes in consultation with the larger study team.

## 3. Results

A total of 123 individuals were invited to participate in an interview approximately two weeks after their results packet had been mailed. A total of 55 agreed to a qualitative interview that was scheduled, on average, 25 days after the results mail-out date (minimum 19 days, maximum 43 days). Five individuals did not participate: two reported not having received their results when contacted; two others did not answer the phone; and one was cancelled due to enrollment limits. Our sample of 50 participants was comprised of equal numbers of males and females ranging in age from 42 to 71 years (mean age 60.9 years). Participants were predominantly white, well educated, and over 50 years old (Table 1).

Interviews ranged from 25 to 100 min in length, with an average length of 46 min. At the time of the interview, no participant had contacted a genetic counselor after receiving their results.

### 3.1. Reactions to Receiving Neutral Results

Almost one-quarter of interviewees reported not having “much of a reaction” (#20: Female, 50), or “emotional response” (#3: Female, 70) to their results. Those who elaborated suggested that because their results were neutral, they were unremarkable: “I don’t know that I had much of a reaction because basically it told me that, uh, it didn’t find any—find any, uh, significant results” (#20: Female, 50). Interviewees “didn’t go have a beer” in celebration (#31: Male, 68), but “just read it and moved on” (#40: Male, 67).

Participants reported a variety of reactions to the letter. Several interviewees described getting neutral results as a “good sign” (#35: Male, 67) and were pleased with the “good news” (#9: Female, 66). Interviewees commonly reported a sense of relief upon receiving such results: “I mean, the—even though I didn’t expect it to say anything […]. I mean it still was kind of a relief, I guess, too, to think—you know, not to have to worry about that or just to know that it was normal” (#52: Female, 42). For some interviewees with a family history of disease, neutral results helped assuage fears. As one participant articulated: “I was kinda relieved that they didn’t find any significant genetic variants, which means, you know, just cause my dad had colon cancer and high cholesterol, doesn’t mean that’s gonna happen to me” (#7: Female, 60).

A few participants reported feeling disappointed by their neutral results. Some participants had hoped their results would explain why certain diseases ran in their families: “I didn’t get any answer on why our family has this issue […]. I was happy I didn’t get any of the 109 results, but I’m still disappointed I didn’t get any answer on why—where does this heart disease come from?” (#48: Male, 63). Other disappointed participants described wishing they had learned something new about their health. One participant described feeling disappointed by her “boring” results and “glad it didn’t cost me [her] any money […] to find out nothing” (#30: Female, 52).

Most interviewees were satisfied receiving neutral results by mail. Some interviewees spontaneously addressed the feasibility challenges of providing face-to-face counseling: “I know you can’t get everybody in to do face-to-face results. So, a letter is absolutely fine” (#24: Female, 61). Several interviewees even articulated a preference for receiving results by mail. They believed that having a letter would help them remember their results because they could review it again as needed.

### 3.2. Understandings of the Results Packet

Interviews explored how participants understood the letter and lab report. Interviewees generally understood the results letter and the limitations that it outlined. By contrast, most participants reported that they did not understand the lab report and were divided as to whether it needed to be included.

#### 3.2.1. The Results Letter

The general “take-away” message from the results letter participants relayed was that no genetic variants were found in their sample: “What I took away is that I was in a study, and they didn’t find anything wrong in the categories that they were studying” (#9: Female, 66). Some participants with the letter in front of them simply quoted the bolded, underlined section of the letter back to the interviewer (see Figure 1). Others simplified the message further to say that “there was no concerns” (#19: Male, 71), or that there was “nothin’ to worry about” (#40: Male, 67). Several interviewees admitted that they “couldn’t remember” getting the letter (#19: Male, 71), or could not “recall exactly what was in it” (#50: Female, 57). Some interviewees described having read enough of the letter to see that “they hadn’t really found anything” (#43: Female, 58) and then either “didn’t read much past that” (#43: Female, 58) or just gave the rest of the letter and the lab report a “quick look over” (#50: Female, 57). A couple of participants could not speak about the letter’s contents with any certainty: “[T]he verbiage in there is over and above my head […]. I’m a truck driver. I’m a union painter. You want me to tell you how to paint your […] house, I can do it. As far as sittin’ and readin’ the letter, I have no idea” (#34: Male, 60).

Interviews explored how well participants understood the limitations of genomic screening enumerated in their results letter (see Figure 1). Some individuals described these limitations unprompted when recounting their results, while others could not recall that the letter included limitations. Many interviewees understood the first limitation to mean that they could still have a genetic predisposition to a disease despite receiving neutral results because not all variants known to cause disease were screened: “I also noted that it—that it didn’t test for everything or every variant” (#47: Male, 59).

Regarding the second limitation, some interviewees explained that the results they received represented “what we know right now, today” (#25: Male, 56), and an updated test might reveal different results: “[T] here may be something in my genes that could be associated with disease. It just—that genetic marker hasn’t been identified yet” (#44: Male, 58). The wording of this limitation led a few participants to believe, incorrectly, that their sample would be “reevaluated at some point in the future” when a “better way of testing” (#27: Female, 51) became available.

Several interviewees interpreted the limitations section of the letter as “lawyer talk” (#36: Male, 53) or a “disclaimer” (#49: Female, 52) written to protect the laboratory or the institution: “Well, that’s standard ‘legalese’ that says, ‘you look good, but if we miss something you can’t sue us ‘cause we told you that we can’t catch everything’” (#25: Male, 56). Nevertheless, interviewees reported the importance of outlining limitations to protect participants from adopting a “false sense of security” about their health (#9: Female, 66). A couple of interviewees mentioned that they would not want individuals who received neutral results to be surprised if they later developed a health problem. In general, participants understood that their sequencing results did not mean that “you’re never gonna acquire any of these diseases […], it’s not a guarantee or anything like that” (#6: Male, 70). Most interviewees expressed the need to “stay on top” of their health, including visiting the doctor and getting regular colonoscopies (#11: Female, 53).

#### 3.2.2. The Lab Report

Several interviewees did not recall receiving the lab report as part of their results packet. Some reported just “glanc[ing] over it” (#11: Female, 53). Those who skimmed the lab report felt that they understood the results letter and did not feel the need to verify their results with the lab report: “I kinda just assumed that the—assumed that the test results would just confirm [the letter], so I didn’t—you know, I don’t think I focused on that a lot” (#6: Male, 70). Almost all interviewees who reported trying to read the lab report found it to be a “mind-boggling” (#8: Female, 68) collection of “scientific mumbo jumbo” (#54: Male, 55). Interviewees frequently stated that they wished it had been written in “layman’s terms” (#18: Female, 69). Some interviewees referenced particular figures and paragraphs in the lab report that they wanted explained. Other participants did not feel the need for clarification because they understood from the cover letter that their results were neutral and the more complicated information featured in the lab report was “for the people doing the research and the people in the medical field that could understand all that stuff” (#7: Female, 60).

While some interviewees felt that the lab report was “a waste of paper” (#38: Male, NA) that they “could’ve lived without” (#16: Male, 56), others saw value in including it with the results letter even if they did not completely understand its content. The report satisfied interviewees’ curiosity about their results, and, for a few, served as proof that their samples were actually studied. One participant noted that the lab report was a critical piece of data supporting the conclusions articulated in the letter: “I think it—the letter would be, uh, purposeless without it. I mean the fact that somebody just says, ‘Okay. You had this, and nothin’ was found, and no further action is needed at this time’—uh, I would’ve been askin’ myself, ‘Why?’” (#28: Female, 60). Some interviewees were happy to have the report because they liked to “have access” to such records at home (#27: Female, 51), and a few noted that it would be handy to have if they ever wanted to bring it along to a medical appointment.

### 3.3. Contextualizing Neutral Genomic Screening Results

Participants incorporated neutral genomic screening results into prior beliefs about their health and the health of their family differently. A couple of interviewees felt that they could no longer “blame” genetics for their health issues: As one father purportedly explained to his children, “You can’t blame your genes anymore. [Laughter] Um, your issues are your own lifestyle, so yes, you can change” (#17: Male, 64). Some interviewees re-interpreted family disease narratives, concluding that their family history of a particular disease was not genetic—as they previously believed—but actually the result of “environmental factors” (#54: Male, 55), “lifestyle” (#2: Female, 67), or “coincidence” (#30: Female, 52). Other participants believed there was a disease-causing variant in their family that had either been missed or not evaluated by the test. The fact that a disease had affected multiple generations, even when people took preventive measures, led a couple of interviewees to conclude that genetics must be involved. As one participant explained.

*“[Y]ou start going back through all your siblings, you go back through your mother’s side of the family back as far as you can and you’ve got the same issue. Okay, you don’t all live the same type of lifestyle. That doesn’t mean that we’re eating a whole beef every day. It doesn’t mean that we’re drinking five gallons of booze a day. It doesn’t mean we’re smoking 20 packs of cigarettes a day. […] What is the reason other than something that’s in our genes that this is getting passed from generation to generation?” (#48: Male, 63)*.

The majority of interviewees did not report a change in their perceived risk assessment for heart disease or colon cancer after learning their results, often citing family history of disease as their explanation. One individual who had had a heart attack and had a family history of heart disease still felt he was at greater risk than the average population because of those factors: “I know that because it runs in my family—I’m probably at higher risk than the average population […] It’s kinda like I, you know, I don’t care if you genetic test me. I know the risks” (#17: Male, 64).

A minority of interviewees thought that their risk for colon cancer or heart disease had lowered as a result of their neutral genetic test results. Several participants had previously considered themselves to be at higher risk of contracting these conditions, but after receiving their results they considered themselves to be at average risk: “[The screening] took me out of the riskier category into the general” (#9: Female, 66). Other interviewees altered their risk assessments more subtly, not going so far as to say their risk was equal to that of the general population, but simply lower than it had been: “So I know that, um, colon cancer runs in the family […] So I know that even though it’s—may not be in the genes, I’m still a high risk for it—but maybe not as high a risk” (#17: Male, 64). Still, even those participants who perceived a shift in risk emphasized their commitment to maintain preventive health measures: “I would say I’m closer to the general population, but it’s not gonna change my preventative pattern of watching for all these problems” (#7: Female, 60).

### 3.4. Misunderstandings about Neutral Screening Results

Some interviewees misunderstood what neutral results meant for their health, forgetting—or not understanding—the limitations outlined in their results letter. Participant misunderstandings of neutral results were largely disparate. A few participants reported a newfound belief that the health condition they had, or that appeared to run in their family, was not genetic: “I guess the research showed that it—that the condition that I have is not, um, genetic” (#53: Female, 69). A couple of participants thought their neutral results extended to family members as well. For example, one participant expressed relief that “there’s no genetic problem, uh, in me or my family” (#44: Male, 58). Other participants mistakenly attributed their neutral genetic sequencing results to their health regimen, preventive care efforts, and even medication compliance: “my, um, Zocor’s doing its job” (#16: Male, 56).

### 3.5. Impact on Health Behaviors

Regardless of how participants interpreted their results, no one disclosed a plan to alter their lifestyle in response to receiving neutral results. The results did not, in their minds, give them a “green flag to be naughty” (#8: Female, 68) or to “eat buckets of fried chicken and ice cream” (#30: Female, 52); although, a couple of interviewees believed other people could misinterpret neutral results that way. In fact, a few interviewees reported that receiving neutral results gave them extra encouragement to maintain a good lifestyle and made them feel “more personal responsibility” (#5: Male, 63) for their health. As one woman articulated, “I guess the results makes me more inclined to work better at a—a better diet and stuff, instead of saying, you know, ‘I can’t help it because it’s genetic,’ you know [Laughter]” (#2: Female, 67).

In general, interviewees reported a commitment to preventive care, including visiting the doctor frequently, keeping up with cholesterol and colon cancer screenings, and taking their medications. Some interviewees attributed their commitment to preventive healthcare measures to a family history of disease or their own health experiences, placing more weight on those considerations than on their genomic test results: “I’ve got polyps, and I’m-I’m—and there’s colon cancer in my family […] with my mom and my dad. And so—you know—I have to get a—the whole deal—the whole colonoscopy and that whole thing” (#13: Male, 59). Many participants also mentioned continued plans to watch their diet, exercise and lose weight, minimize smoking and drinking, and avoid pesticides to protect their health. Although receiving neutral results appeared to have a minimal effect on interviewees’ plans to maintain good health, some admitted that there was always room for improvement and they could “probably do better” (#53: Female, 69) in their efforts to stay healthy.

## 4. Discussion

The findings we describe suggest that reporting neutral genomic screening results by mail is unlikely to cause distress or result in harm as a result of misunderstandings about test results. We found that the majority of individuals understood their results and were satisfied receiving those results by mail. Moreover, our findings suggest that receiving neutral results by mail, even in the absence of genetic counseling, is unlikely to lead to unhealthy behavioral changes. These findings are encouraging and support two-tiered reporting strategies that help genetic counselors spend more time with individuals who receive positive genomic test results while using other communication types to report neutral screening results. As other institutions consider approaches to reporting neutral genomic screening results, our findings highlight several practical considerations in reporting results by mail.

First, genomic sequencing programs should work to ensure neutral results are presented in a clear, easy-to-read, easy-to-understand format. Although many participants reported difficulty understanding the laboratory report and expressed an interest in consulting a healthcare specialist about its meaning—not unlike patients described in a study by Phelps and colleagues who wanted additional information about their results, particularly the lab report—that [18] “interest” never translated into calls with genetic counselors [11]. If lab reports are going to be included with a results letter, programs should work with sequencing laboratories to make those letters more accessible. To assist in those efforts, Stuckey et al. provide insights into parents’ values regarding their child’s whole genome sequencing lab report [19] and Haga et al. outline for consideration four different “patient-friendly” laboratory report formats [20].

Second, our findings highlight the need to contextualize neutral results within the broader context of other health messages recipients might be receiving. Our participants reported a range of emotional reactions to receiving neutral genomic results. Many described a sense of relief upon learning their results, a reaction reported in other studies [21,22,23,24]. Still, many of our participants, like those described by Michie and colleagues, were not reassured by their results [25]. For example, some participants wondered how a particular disease could run in their family if the test results did not identify a genetic risk factor: a lingering question in the minds of participants in other studies as well [23,24,26]. Individuals’ personal experiences of disease shape how they interpret genetic risk [22]; and those with a family history of disease may react very differently to neutral genetic test results than persons without a family history [23]. Moving forward, genomic screening letters may need to explain more explicitly how one can have a family history of disease and nonetheless receive a neutral genomic screening result.

Third, our findings suggest that neutral results should also include a clear explanation of whether a person’s risk for disease has changed. We observed considerable variability in participants’ appreciation of the limitations of genomic screening. As reported by Butterfield and colleagues [27], many of our participants missed the “nuance” of their neutral genomic screening result, broadly interpreting it as “good news”. Some participants felt that their risk of disease had lowered, while others concluded that a familial disease must not be genetic in origin [26]. These findings are consistent with misunderstandings reported in other screening contexts, such as cancer screening [28] and non-invasive prenatal testing [29,30].

Effectively communicating neutral results in writing will be a critical challenge for genomic screening. While it is impossible to prevent all misunderstandings, future research exploring alternative mechanisms for reporting neutral results might consider addressing the issue of risk more explicitly. As risk for the conditions examined is polygenic, complex, and influenced by gene–environment interactions, future studies reporting neutral results might consider telling neutral results recipients that their personal risk of developing a disease has likely not changed and stressing the importance of following established preventive health measures. Genetic counselors, clinical geneticists, study coordinators, and community/patient advisory boards—in collaboration with cultural and linguistic specialists—should develop neutral results communications that are scientifically accurate, clear, and appropriate for participants from diverse backgrounds and communities. Formally evaluating the effectiveness of these communications prior to mass distribution can also help identify areas of confusion and provide additional opportunities to revise the results letter and/or accompanying information with the goal of avoiding potential misunderstandings of these results.

Fourth, concerns that recipients of neutral genomic screening results may abandon their healthy lifestyles in response to what they see as “good news” may be unfounded, at least in populations similar to ours. Participants did not reveal plans to change health behaviors in ways that might be concerning to healthcare providers. This may be attributable to the fact that individuals pursuing genomic screening are often committed to being vigilant about their health [21,26]. Similar results have been reported in the context of cancer screening, where several studies—but not all [31]—have reported that individuals who received neutral screening results remained interested in pursuing additional clinical evaluations [32,33]. The endurance of this health vigilance highlights the possibility that perceptions of disease risk may not be easily changed regardless of new information [34].

### Limitations and Future Research

A significant study limitation was the homogeneity of interviewees, who were mostly older, white, well-educated, financially comfortable individuals living in Minnesota [12]. Given the composition of Mayo Clinic Biobank participants, we were limited in our ability to create a demographically diverse sample. Another potential limitation of our study was that half of the interviewees had their results letter accessible during the interview, which may have introduced bias in how they responded to questions. Additionally, interviewees often discussed the results letter and lab report interchangeably, making it difficult to discern the source of specific misconceptions about their results. Since genomic screening was provided within the context of a translational research study, participant responses also may not reflect attitudes of patients receiving neutral genomic screening result in clinical settings. Lastly, whether our approach to disclosing neutral results affected participants’ comprehension and interpretation remains unclear. A different results letter (e.g., one that included explicit explanations about participant risks), a different communication modality (e.g., via a patient account or email), or different supporting materials (e.g., a frequently-asked-questions brochure) could have produced different participant responses, particularly with respect to understanding of the neutral results.

Future research should seek to identify predictors of poor understanding of neutral genomic screening results and ways to identify individuals who might benefit from genetic counseling support [11]. Additionally, comparative effectiveness studies of different ways of communicating neutral results would be useful [18]. Studies comparing communications by mail, email, text, telephone, and in-person encounters, for example, could assess trade-offs associated with different modes of communication and different degrees of genetic specialist involvement. Since the results of such studies will likely differ based on the sample populations, comparative effectiveness studies will need to include representation from diverse communities. As we continue reporting neutral results to participants and patients, consideration might need to be given to hybrid approaches to the return of neutral results processes depending on the needs of the community: combining bilingual written results with follow-up calls, for example, as was done in a recent study at a federally qualified health center in Arizona [35] might be one way to mitigate potential for misunderstandings. Engaging in community-based research with linguistic and cultural specialists included as part of the team might also help ensure that whatever written communication is provided around neutral results is done so in a culturally meaningful way using language appropriate for a range of literacy levels.

## 5. Conclusions

Our results suggest that individuals value receiving neutral genomic screening results and are satisfied receiving those results by mail. Reporting such results by mail will most likely not cause widespread anxiety, prompt individuals to call their healthcare providers for interpretive assistance, or generate misunderstandings that could reduce compliance with preventive health recommendations. Nonetheless, some people may benefit from additional genetic counseling support to ensure understanding of neutral results and situate those results within the context of unique concerns, family histories, and prior illness experiences.

## Figures and Tables

**Table 1:** Characteristics of individuals who received a neutral genomic screening report and participated in an in-depth interview.

Characteristic	N (%)
**Sex**	
Male	25 (50)
Female	25 (50)
**Age (years) at Study Invitation**	
Mean (SD)	60.9 (6.9)
Range	42–71
**Race**	
White	49 (98)
Native American	1 (2)
**Education**	
High school graduate	5 (10)
Some college	18 (38)
College graduate	17 (35)
Graduate school	8 (16)
Other	1 (2)
**Last Checkup**	
Within past year	37 (74)
Within past 2 years	7 (14)
Within past 5 years	4 (8)
5 or more years ago	2 (4)
**Genetic Knowledge** [16]	
Mean (SD)	7.9 (2.3)
Range	0–11
**Health Literacy** [17]	
Adequate	46 (92)
Inadequate	4 (8)
**Insurance Coverage**	
Employer	38 (78)
Public program	16 (33)
Private	2 (4)
Uninsured	0 (0)

## Data Availability

Qualitative data for this study have not been made publicly available.
